# The Synthetic Antimicrobial Peptide Pexiganan and Its Nanoparticles (PNPs) Exhibit the Anti-*Helicobacter pylori* Activity *in Vitro* and *in Vivo*

**DOI:** 10.3390/molecules20033972

**Published:** 2015-03-02

**Authors:** Xiao-Lin Zhang, An-Min Jiang, Zhong-You Ma, Xian-Bao Li, You-Yi Xiong, Jin-Feng Dou, Jian-Fei Wang

**Affiliations:** 1 The Department of Pharmacy, Food and Drug School, Anhui Science and Technology University, Fengyang 233100, China; E-Mails: mazy@ahstu.edu.cn (Z.-Y.M.); lixb@ahstu.edu.cn (X.-B.L.); xyytc1@163.com (Y.-Y.X.); doujf@ahstu.edu.cn (J.-F.D.); 2 The School of Life Science, University of Science and Technology of China, Hefei 230032, China; E-Mail: SA12226011@mail.ustu.edu.cn; 3 The Ministry of Agriculture Key Laboratory of Microbial Organic Fertilizer, Bengbu 233030, China

**Keywords:** antimicrobial peptide, pexiganan, nanoparticles, *Helicobacter pylori*

## Abstract

The aim of this study was to probe the potential anti-*H. pylori* activity of the synthetic antimicrobial peptide pexiganan, which is an analog of the peptide magainin, and its nanoparticles (PNPs) that were prepared in our laboratory. To compare their antibacterial effects *in vitro* and *in vivo*, studies of *H. pylori* growth inhibition, kinetics and resistance assays were undertaken. The gastric mucoadhesive efficiency and *H. pylori* clearance efficiency of pexiganan and PNPs were evaluated in rats and mice infected with *H. pylori*. The eradication of *H. pylori* was determined using urease tests and a microbial culture method. We observed that PNPs adhered to gastric mucosa more effectively owing to a prolonged stay in the stomach, which resulted in a more effective *H. pylori* clearance. In addition, PNPs had greater anti-*H. pylori* effect than pexiganan in infected mice. The amount of pexiganan required to eradicate *H. pylori* was significantly less using PNPs than the corresponding pexiganan suspension. The results confirmed that PNPs improved peptide stability in the stomach and more effectively eradicated *H. pylori* from mice stomachs than pexiganan.

## 1. Introduction

*H. pylori* is a Gram-negative microaerophile bacillus that often infects humans during childhood. It colonizes gastric mucosa of nearly 50% of the world’s population and, in some cases, for life. *H. pylori* plays a major role in most cases of gastric duodenal ulcers, gastritis, and gastric cancer [1], therefore, eradicating *H. pylori* infection is a very valuable strategy for curing a gastric or duodenal ulcer and is also likely to be a promising approach to prevent the occurrence of gastric cancer [2]. Triple therapy, which combines amoxicillin, metronidazole or clarithromycin and a proton pump inhibitor or stomach bismuth, is now frequently used to treat gastroduodenal disease associated with *H. pylori* infection in clinical settings. However, eradication is sometimes unsuccessful. In addition, the high cost of therapy, harmful side effects, and poor treatment compliance due to patients’ lack of willingness to take so many different drugs are major drawbacks in the treatment of *H. pylori* infections [3,4]. Recently, a decline in the treatment efficacy of standard triple therapy has been reported. Treatment failure is generally due to increased antimicrobial drug resistance [5]. Because drug resistance is increasing in *H. pylori*, newer therapeutic strategies must be implemented as quickly as possible. One study reported that *H. pylori* eradication would be more successful if sensitivity testing were to be performed before treatment [6]. This would allow the selection of antibiotics according to their susceptibility of the strain of *H. pylori* to be eradicated. Antimicrobial peptides (AMPs) rapidly kill microbes and have broad spectrum antimicrobial activity. In particular, they are not affected by classical mechanisms of antibiotic resistance [7,8,9]. These are potential therapeutic advantages in the treatment of *H. pylori* infection. It is an essential requirement that an antimicrobial agent must have selective toxicity to the microbial target. AMPs have this important feature, preferentially interacting with microbial cells but are usually non-toxic to mammalian cells at antibacterial concentration. Interestingly, although antimicrobial activity is the fundamental biological role of AMPs, recent studies have further elucidated some novel functions of these molecules, including wound healing, neutralization of endotoxins, immunomodulatory activities, and anti-neoplastic properties [10,11,12,13,14,15]. In addition to these advantages of AMPs, it is a promising candidate for topical application in the treatment of *H pylori* infection, yet its comprehensive study has not been reported. In this study, we treated *H. pylori* infection using an AMP consisting of a 22-amino-acid peptide called pexiganan, which is a magainin AMP analog isolated from the skin of the African clawed frog. It has exhibited broad-spectrum antibacterial activity *in vitro*, and it has been tested against 3109 clinical isolate strains of Gram-negative and Gram-positive, aerobic and anaerobic bacteria [16]. This is the first study of its activity against *H. pylori* activity *in vitro* and *in vivo*.

Another reason for the incomplete eradication, and/or difficult in eradicating *H. pylori* is the low antibiotic concentration in gastric mucosa. The bacteria stay under the mucosa where antibiotic concentrations are low. In addition, in the low pH of the gastric fluid, antibiotics are unstable. As a result of gastric emptying, the residence time is short for antibiotics in the stomach [3]. A strategy for more effective delivery of antibiotics to *H. pylori* is mucoadhesive nano or micro particulate mucosal delivery systems. These systems, based on the mucoadhesive delivery of particulates, remain in stomach mucosa longer and thus are beneficial for local delivery of the antibiotic and a longer retention time in the gastric mucosa [17]. Most mucoadhesive delivery systems are prepared using positively charged polymer materials such as chitosan [18,19], or chitosan-coated sodium alginate [20]. These delivery systems provide an intimate contact with the negatively charged gastric mucosa glycoprotein and the sialic acid, sulphate groups or carboxyl in the mucus. Additional interaction with the mucosal membrane may occur via electrostatic attraction, polyvalent adhesive interaction, van der Waal forces and H-bond formation [21]. The system also has the advantage of offering effective drug penetration across the mucus layer and protecting acid-sensitive drugs from degradation. This nanoparticle gastric mucosa-specific delivery system also would significantly increase the gastric mucus membrane residence time of the drug, decrease the diffusional distance, effectively increase the local drug concentration, minimize the resistance problems associated with topical low drug concentration and close windows of mutations. The main purpose of this study was to more comprehensively evaluate the effectiveness of pexiganan and pexiganan chitosan-alginate polyelectrolyte complex nanoparticles (PNPs) for in an *in vitro H. pylori* growth inhibition study, as well as kinetics studies, and resistance assays and *in vivo* mucoadhesion efficiency and *H. pylori* eradication therapy in infected mice.

## 2. Results and Discussion

### 2.1. MICs

As shown in Table 1, the two *H. pylori* clinical isolate strains and the standard strain were sensitive to pexiganan and PNPs. These isolates and the standard strain were effectively inhibited by pexiganan and PNPs at a concentration of 4 µg/mL. This result showed that the MIC of pexiganan and PNPs for *H. pylori* is 4 µg/mL.

**Table 1:** MICs of pexiganan and PNPs for a *H. pylori* gastric ulcer strain, gastric cancer strain and standard strain (ATCC 43504).

Clinic Strains	MIC (µg/mL)		
	Pexiganan	PNPs	Placebo Nanoparticles
Gastric ulcer strain	4	4	ND
Gastric cancer strain	4	4	ND
ATCC 43504	4	4	ND

PNPs: containing the amount of pexiganan, ND: the MIC could not be determined.

### 2.2. In Vitro Antibacterial Activity

**Figure 1:**
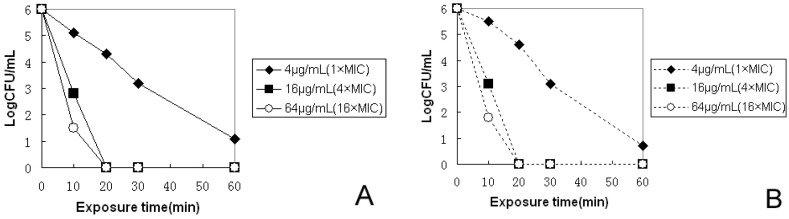
Bactericidal kinetics study. The bactericidal activity of pexiganan (**A**) and PNPs (**B**) against *H. pylori* were monitored for the first 1 h. After 0, 10, 20, and 60 min of exposure time at 37 °C, aliquots were diluted (serial 10-fold dilutions) and plated for CFU counts after 72 h incubation at 37 °C.

The kinetics of bactericidal activity against two *H. pylori* strains were investigated by counting CFUs after bacterial exposure to pexiganan or to PNPs at three different concentrations. Cell viability was measured within the first 60 min at different time points. As shown in Figure 1, for the *H. pylori* clinical strain, the bactericidal effect was dependent on drug concentrations of pexiganan and PNPs. To confirm that the best bactericidal activity against *H. pylori* was more than 10^6^ CFU/mL *H. pylori w*ere eradicated within 20 min at 16 µg/mL (4× the MIC) of pexiganan and PNPs. The results indicated that *H. pylori* could be rapidly killed at appropriate concentrations of pexiganan and PNPs, thus, they are excellent bactericidal agents for *H. pylori*. To further confirm the bactericidal effect, bacterial were cultured for up to 72 h, and monitored. No regrowth was observed and no colonies of *H. pylori* were present after 0.2 mL of the cultures was plated for 72 h.

### 2.3. In Vitro Resistance Studies

**Figure 2:**
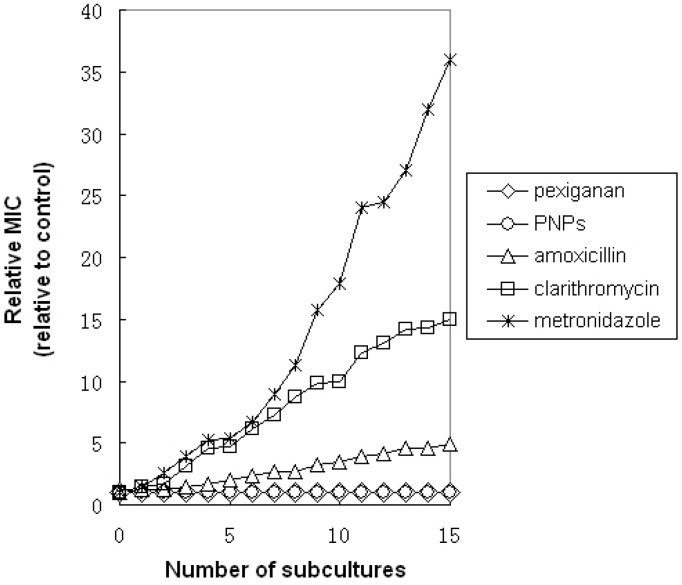
*In vitro* development-of-resistance studies. Evolution of MICs after successive exposures of *H. pylori* to subinhibitory concentrations of the antimicrobial agent. After 15 serial passages, the relative MIC was the normalized ratio of the MIC obtained for a given subculture to the MIC that obtained for first-time exposure.

To assess whether emerging resistance could be developed using multiple exposures of *H. pylori* to pexiganan and PNPs, the clinical isolate strains of *H. pylori* were cultured for 15 consecutive generations in the presence of pexiganan and PNPs. As shown in Figure 2, after 15 consecutive subcultures, we found that the relative MIC of pexiganan and PNPs against *H. pylori* remained stable. 

In contrast, we found that the MIC gradually increased for metronidazole, clarithromycin and amoxicillin during the same period of time, indicating the emergence of resistant bacteria. At the 15th generation, we found that the MICs of these drugs increased by 36-fold for metronidazole, by 15-fold for clarithromycin, and by 6-fold for amoxicillin. Moreover, in the 15th generation of *H. pylori* that had developed resistance to antibiotics, the *H. pylori* were still susceptible to pexiganan and PNPs, and their MICs were similar to those at the beginning. The results showed that the pexiganan and PNPs could be used to treat *H. pylori* infection that is resistant to some antibiotics.

### 2.4. In Vivo Bioadhesivity

**Figure 3:**
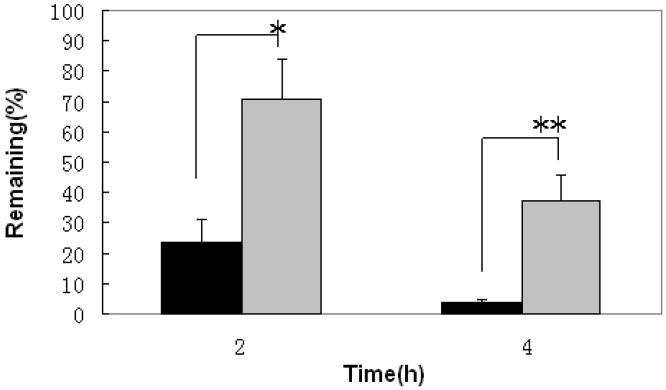
The examination of gastric bioadhesive property. The remaining percentage of pexiganan on the gastric mucosa of rats 2 h (* *p* < 0.05) and 4 h (** *p* < 0.01) after oral administration of pexiganan suspension (black bars) and pexiganan nanoparticles (PNPs) (gray bars) (*n* = 5).

The *in vivo* gastric bioadhesive property was examined using uninfected rats under fed conditions. The remaining percentage of pexiganan 2 and 4 h after pexiganan suspension and PNPs administration, 70.6% ± 13.2% and 37.1% ± 7.8%, respectively, was approximately three times (*p* < 0.05) and ten times (*p* < 0.01 ) higher than after pexiganan suspension administration, 23.5% ± 8.5% and 3.6% ± 1.3%, respectively (Figure 3). The PNPs formulation had highest bioadhesive value. This study suggests that PNPs (chitosan-alginate polyelectrolyte complex) present on cationic amino groups can interact with mucin glycoproteins, sialic acid and other anionic moieties present on gastric mucosa [22], adhering to the mucosa more strongly and increasing the stability of pexiganan. In addition, these products could stay in the stomach for a prolonged period for more effective *H. pylori* clearance.

### 2.5. In Vivo H. pylori Clearance Efficiency

**Figure 4:**
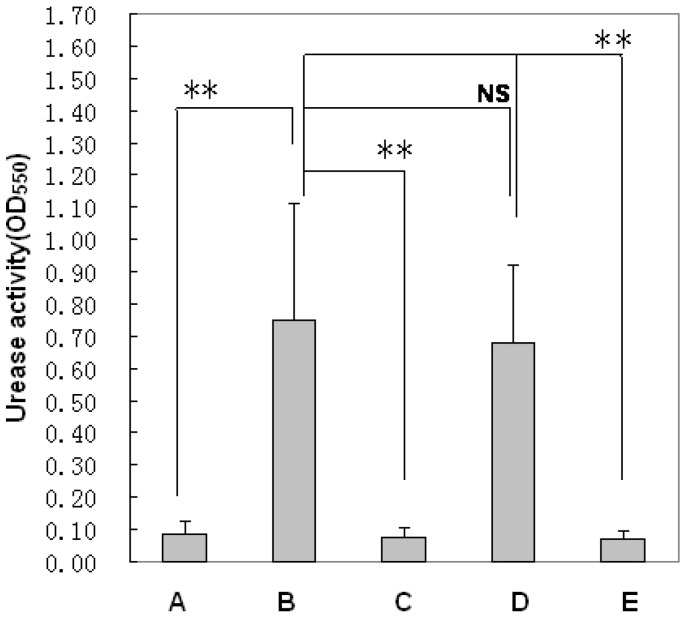
Evaluation of clearance efficiency of *H. pylori* infection based on urease activity tests. (A) The normal control mice; (B)The model mice of *H. pylori* infection; (C) The mice treated with pexiganan; (D) The mice treated with placebo nonaparticles (control); (E) The mice treated with PNPs. (** *p* < 0.01 and NS: no significant differences).

An *H. pylori* clearance study of pexiganan and PNPs was carried out with a mouse model infected with human *H. pylori in vivo*. Pexiganan and PNPs were orally administered at doses of 1, 3, 10 or 30 mg/kg once every day for three consecutive days to *H. pylori*-infected mouse stomachs. As shown in Figure 4, the results of the urease test showed significant differences between the pexiganan and PNPs groups. It is obvious that the *H. pylori* urease activities were lower in mouse stomachs treated with PNPs than pexiganan (*p* < 0.01).

Table 2 shows the mean bacterial count in mouse stomachs, the control group mice receiving no drug, and the average about 10^7.87^ (CFU/stomach) bacteria colonized in each mouse stomach. The mean bacterial count of the mouse stomachs treated with oral pexiganan decreased as the dose increased, but complete clearance was not observed, even the highest dose of 30 mg/kg. 

**Table 2:** Effect of repetitive administration of pexiganan and PNPs against gastric infection caused by *H. pylori* in mice.

Formulations	Dose (mg/kg)	Clearance Rate (No of Mice Cleared Infection/Total No) (%)	Bacterial Recovery (Log CFU/Stomach)
Vehicle control Pexiganan (peptide)	0	0/6(0)	7.87 ± 0.83
	1	0/6(0)	7.62 ± 0.61
	3	0/6(0)	5.87 ± 0.52
	10	0/6(17)	3.29 ± 0.37
	30	3/6(50)	1.86 ± 0.28
Placebo nanoparticles (control) PNPs	0	0/6(0)	6.73 ± 0.54
	1	1/6(17)	4.18 ± 0.93
	3	4/6(67)	1.57 ± 0.78
	10	6/6(100)	ND
	30	6/6(100)	ND

PNPs: containing the amount of pexiganan, ND: the bacteria colonies could not be determined.

However, the mean bacterial count after three consecutive days of treatment with PNPs containing pexiganan at a dose of 1 mg/kg revealed that approximately 17% of *H. pylori* was inhibited by PNPs, almost equal to that of pexiganan at the dose of 10 mg/kg, a dose significantly (*p* < 0.01) lower than that of pexiganan. This result clearly shows that, owing to the gastric mucosa specific delivery of PNPs, it is a very effective treatment for the clearance of *H. pylori* at a much lower dose than pexiganan. Under *H. pylori* treatment with PNPs at the same doses of pexiganan (10 and 30 mg/kg), the bacteria were completely cleared from the stomachs and no *H. pylori* colonies were detected in the gastric samples. The results indicated that a 100% clearance rate was obtained at the doses of 10 and 30 mg/kg. In addition, 67% of inhibition was obtained with PNPs containing pexiganan at a dose of 3 mg/kg, and the *H. pylori* bacterial load was dramatically reduced in the stomachs. The results demonstrated that PNPs have a stronger clearance efficiency for *H. pylori in vivo* and can be used to treat *H. pylori* infection.

For a comprehensive study of pexiganan and PNPs against *H. pylori*, the MICs of pexiganan and PNPs for *H. pylori* were determined *in vitro*. The MICs of pexiganan for *H. pylori* were 4 μg/mL (two clinical strains of *H. pylori* were isolated from patients with gastric cancer and gastric ulcer, and a standard ATCC 43504 served as the control) (Table 1). The kinetics of their bactericidal activity were studied. At 16 μg/mL, colony forming units were reduced to zero at 20 min for pexiganan and PNPs (Figure 1)*.* These results revealed a rapid onset of killing *H. pylori* by pexiganan and PNPs *in vitro*. Attempts to generate resistance to *H. pylori* by repeated treatment under the conditions of subinhibitory concentrations of pexiganan and PNPs were unsuccessful (Figure 2). However, *H. pylori* quickly developed resistance to several other antimicrobial compounds, including metronidazole, clarithromycin and amoxicillin, consistent with a recent report [23]. Moreover, those bacteria of *H. pylori* that were resistant to antibiotics remained susceptible to pexiganan and PNPs. Much below the MIC, a quick onset of activity and a low propensity of generating resistance make pexiganan and PNPs ideal drugs or the treatment of *H. pylori* infection. The potential toxicity of pexiganan has been investigated systematically by measuring the peptide’s hemolytic activity in human red blood cells in other studies. The reports suggested that at least 250 μg/mL is necessary to induce 100% hemolysis [24,25,26]. Two phase III clinical trials have reported that no adverse side effects have been found for pexiganan [27]. The antimicrobial mechanism of pexiganan has been studied by others, who found that the cell membrane was disrupted by pexiganan, as determined by monitoring the leakage or uptake of the peptide’s fluorescent labeling molecules. The cell membranes were affected within 5 min of exposure to pexiganan, indicating that pexiganan rapidly disrupts membranes [28]. The effect of pexiganan on the lipid bilayer demonstrated the mechanistic route of cell membrane disruption [29]. The detergent behavior of pexiganan and its peptide-induced disorders of the lipid bilayer in the hydrophobic region were studied using solid-state NMR by others, revealing that cell membrane was disrupted by pexiganan [30]. These results confirm the reason for the quick onset of activity and low propensity for resistance to pexiganan in our study. These promising attributes are useful for developing pexiganan as an anti-*H. pylori* infection agent.

*H. pylori* has evolved a unique way to survive in the harsh acidic gastric environment by colonizing the depth of the gastric mucosa and adhering to mucosal epithelial cells using flagella to adhere and penetrate the mucosal layer [31]. Gastric motility and emptying are associated with high mucus turnover, which is associated with a shorter gastric residence for drugs. Moreover, the gastric proteolytic activity degrades and inactivates protein drugs [32]. To overcome these limitations, PNPs are particulate mucosal membrane drug delivery systems that penetrate the mucosal membrane in close proximity to the infection site of *H. pylori*. We present first evidence that mucoadhesive PNPs are usefulness for the eradication of *H. pylori* the *in vivo*. The evaluation of PNPs against *H. pylori* was carried out in mice infected with human clinical stains. The results showed that there was a discrepancy of treatment for *H. pylori* between pexiganan and PNPs (Table 2). The reasons for the lower antibacterial effect for pexiganan are: (i) accelerating the degradation of pexiganan owing to the low pH of gastric fluid and the presence of active pepsin at stomach that can cleave peptide bonds between amino acids such as phenylalanine, tryptophan, and tyrosine; and (ii) the stomach residence time is shorter after oral administration with the conventional dosage form of pexiganan due to gastric emptying [33]. Moreover, to inhibit *H. pylori*, bactericidal concentrations of pexiganan should be availablearound the gastric epithelial cells and mucusosal layer where *H. pylori* resides [34]. However, the concentrations of pexiganan for bactericidal activity are difficult to achieve under the gastric mucosal layer. Thus, the shorter duration of contact with the gastric mucosa using the conventional dosage form of pexiganan results in an incomplete eradication of *H. pylori* (Table 2). At the same time, we found that PNPs remained on the gastric mucosa for a longer time around the gastric mucosa than pexiganan administered in the form of a suspension (Figure 3). The PNPs provided a greater scavenging activity for *H. pylori* than the pexiganan suspension. In conclusion, pexiganan is a promising anti-*H. pylori* agent and more effectively clears *H. pylori* In PNPs than pexiganan administered in a suspension form (Figure 4 and Table 2). The use of mucoadhesive PNPs may lead to the eradication of *H. pylori* infection in humans.

## 3. Experimental Section

### 3.1. Agents

Pexiganan, also called MSI-78, consisting of 22 amino acids (Gly-Ile-Gly-Lys-Phe-Leu-Lys-Lys-Ala-Lys-Lys-Phe-Gly-Lys-Ala-Phe-Val-Lys-Ile-Leu-Lys-Lys-NH_2_) was synthesized at Sangon Biotech Co., Ltd. (Shanghai, China). A chitosan-alginate polyelectrolyte complex pexiganan nanoparticles (PNPs) for delivery of pexiganan were prepared in our laboratory using a modified ionic gelation method [35,36], Influence the characteristics of nanoparticulate delivery systems were optimized according to concentrations and ratio of polymer, chitosan (degree of deacetylation 80.0%–95.0%, viscosity 50–800 mPa.s) and sodium alginate (viscosity, 1.05–1.15 Pa.s) were purchased from Sinopharm Chemical Reagent Co., Ltd. (Shanghai, China)/peptide purity (≥95%)/surfactant (Pluronic F-127, BASF, Ludwigshafen, Germany), mixing time and speed, pH, homogenization speed and time *etc.* Among prepared PNPs, a pexiganan nanoparticle with a paticle size of 415 ± 26 nm, a Zeta potential of 47.78 ± 3.5 mV and an encapsulation efficiency of 82.8% ± 4.5% was used in this study. Brain heart infusion (BHI) broth and agar for *Helicobacter pylori* culture were purchased from Hope Biol-technology Co., Ltd. (Qingdao, China). Defibrinated sheep blood was purchased from Jiulong Biological Products Co., Ltd (Zhenzhou, China). Fetal calf serum (FCS) was purchased from Sangon Biotech Co., Ltd. An MGC Anaero Pack and an anaerobic jar were purchased from Mitsubishi Gas Chemical Company, Inc. (Tokyo, Japan).

### 3.2. Animals

Six-week-old male Kunming mice and 200–300 g male Sprague-Dawley rats were purchased from Changlinhe Pharmaceutic Co., Ltd. (Anhui, China). The animals were housed in polycarbonate cages under standard laboratory conditions and fed a SPF grade commercial pellet diet with sterile water ad libitum. All procedures were conducted according to the P.R. China legislation No. 8910 M047 covering the care and use of laboratory animals and in accordance with the guidelines established by the Institute for Experimental Animals of Anhui Science and Technology University. The procedures were approved by the university ethics committee for animal experiments.

### 3.3. In Vitro Growth Inhibition Studies of pexiganan and PNPs

Two clinical *H. pylori* strains were isolated from two patients with gastric cancer and gastric ulcer, obtained from the Jiangsu University (Zhengjiang, China). The *H. pylori* (ATCC 43504) strain was preserved in our laboratory. Antibacterial activity was evaluated using a rapid screening analysis for growth inhibition in BHI broth supplemented with 10% FCS, amphotericin B (5 μg/mL), trimethoprim (5 μg/mL), vancomycin (10 μg/mL) and cefsulodin (5 μg/mL). The bacterial numbers were estimated using optical density measurement methods at 600 nm and comparison to a calibration curve. The minimum inhibitory concentration (MIC) was detected using a 100 µL bacterial suspension (10^6^ bacteria/mL at the exponential phase of growth) added to 100 µL through two-fold serial dilutions of pexiganan peptide or its nanoparticle test compound concentrations (initial concentration of 32 µg/mL) and culture medium without the test compound. Samples were placed in 96-well plates using three parallel control. The inhibition of bacterial growth was assessed through optical density measurement methods (at 600 nm) after a culture period of 24 h with shaking at 200 rpm under microaerophilic conditions (MGC Anaero Pack in anaerobic jar, Mitsubishi Gas Chemical Company, Inc.) at 37 °C. The MIC was determined as the minimal concentration able to inhibit visible growth.

### 3.4. In Vitro Kinetics Studies of Pexiganan and PNPs

According to the encapsulation efficiency of the PNPs, 100 µL stock solutions of pexiganan and PNPs were prepared in culture medium to obtain a final concentration of 4 µg/mL, 16 µg/mL or 64 µg/mL pexiganan with three parallel control. Then, 100 µL of *H. pylori* (10^6^ CFU/mL at the exponential phase of growth) was added to the solutions. After 0, 10, 20, 30, and 60 min of culture under microaerophilic conditions at 37 °C, the cultures were serially diluted up to 1/10,000 by the addition of 20 µL of culture to 180 µL of BHI broth. The samples were thoroughly mixed and each 50 µL dilution was plated on BHI agar containing 7% sheep blood plate for CFU counts after 48 h of culture under microaerophilic conditions at 37 °C.

### 3.5. In Vitro Resistance Assays of pexiganan and PNPs

For the resistance studies, the two clinical *H. pylori* strains described above were used. In this study, all bacteria were at the exponential phase of growth. The MIC for an antimicrobial agent was determined as above. Following an overnight culture, bacteria were gathered from culture wells. In the samples near 50% growth inhibition, the bacteria were washed and diluted using fresh culture medium and then cultured overnight. MIC determinations were made using 15 similar serial passages. During these subcultures, the MIC was assessed and compared with every new control generation, which consisted of bacteria cultured in culture wells without antimicrobial agents from the previous generation. The relative MIC of each experiment was calculated according to the ratio of MIC obtained from a given subculture to that obtained of the initial exposure. The statistical data were processed from three repeats performed in triplicate. A similar approach was also used for metronidazole, clarithromycin and amoxicillin as the control.

### 3.6. Evaluation Gastric Mucoadhesion of pexiganan and PNPs

The rats were fasted for 24 h before the experiments but were allowed free access to water. PNPs or pexiganan were suspended in PBS (pH 7.4) at a final concentration of 1 mg/mL. The solution was orally administered to mice at a 10 mg/kg body weight dose of pexiganan. Pexiganan and PNPs solutions were administered following a published procedure [37]. Pexiganan and PNPs could be attached to the gastric mucosa of conscious rats. At 2 or 4 h after oral administration, the rats were euthanized under ether anesthesia. The stomachs were excised and dissected. The amount of pexiganan associated with the gastric mucosa was evaluated according to the following method. The stomachs were carefully washed 3 times with 30 mL PBS. The washed solution samples were centrifuged (pexiganan samples at 2500× *g* for 5 min to collect the supernatant , PNPs samples at 8600× *g* for 10 min to collecte the PNPs precipitate. Then the PNPs precipitate were digested in 1% acetic acid for 20 min at 37 °C for complete digestion to release pexiganan, and then centrifuged at 2500× *g* for 5 min to collect the supernatant). The supernatant was examined using the BCA Protein Assay Kit (Sangon Biotech Co., Ltd.). The amount of pexiganan is relative to standard protein amount according to the method of BCA Protein Assay Kit introduction. The gastric mucoadhesion efficiency was calculated using the following equation: Gastric mucoadhesion % = [(the amount of pexiganan detected in supernatants)/(the total amount of pexiganan added) × 100].

### 3.7. In Vivo H. Pylori Clearance Study of pexiganan and PNPs

An animal model of *H. pylori* infection was established using the two clinical strains. Six mice in each group were randomly assigned to 10 groups and were gavaged with 0.3 mL BIH broth containing 10^8^ CFU of *H. pylori* after fasting for 24 h. Fourteen days after inoculation, pexiganan and PNPs were orally administered once a day for three consecutive days at a dose of 1, 3, 10 or 30 mg/kg body weight. Placebo mucoadhesive nanoparticles were orally administered in the same manner to act as a control. One day after final administration, the mice were euthanized under anesthesia and the stomachs were homogenized in 1.5 mL BHI broth with a tissue homogenizer. For the urease tests, 0.1 mL gastric homogenate was added to 3 mL urea (1 mg/mL peptone, 1 mg/mL glucose, 5 mg/mL NaCl, 2 mg/mL KH_2_PO_4_ and 1% urea) that contained 0.25% phenol red (at least three repeats). The reaction were allowed to proceed for 4 h at 37 °C. Gastric bacteria urease activity was measured by spectrophotometry at OD_550_nm. *H. pylori* infection was quantified according to absorbance values after urea hydrolysis. The eradication of the infection from animals was defined as the values of absorbance at 550 nm within two standard deviations of the mean *versus* uninfected control mice [38]. In addition, the eradication of *H. pylori* was determined using a microbial culture method. The remaining homogenate was 10-fold serially diluted and plated on BHI agar plates supplemented with 7% sheep blood and antibiotics as above. The plates were incubated under microaerobic conditions for 4 d at 37 °C. The number of *H. pylori* bacteria was determined by counting bacterial colonies on the agar plates (at least in triplicate). The bacteria were further identified via microscopy, an oxidase test, a catalase test and a urease test, according to published methods [39]. The number of bacterial colonizations per mouse stomach was calculated by counting the colonies per plate and were expressed as log CFU per gastric sample.

### 3.8. Statistics

The difference was statistically analyzed between the control group and the pexiganan or PNPs-treated groups, and between the pexiganan-treated group and the PNPs-treated groups. Stomach bacterial counts were statistically analyzed using one-way analysis variance and Dunnett’s multiple comparison test. The significant differences between different groups were defined as *p* < 0.05.

## 4. Conclusions

The synthetic antimicrobial peptide pexiganan and its nanoparticles (PNPs) possess anti *Helicobacter pylori* infection activity. The *in vitro H. pylori* growth inhibition studies, kinetics studies and resistance assays were studied through comparison of pexiganan to PNPs. The MIC of pexiganan for *H. pylori* is low (4 µg/mL). The bactericidal activity kinetics against *H. pylori* clinical strains were studied at 16 µg/mL. Colony forming units were reduced to zero after 20 min by pexiganan and PNPs*.* These results show the rapid onset of activity for killing *H. pylori* displayed by pexiganan and PNPs *in vitro*. Attempts to induce resistance for *H. pylori* showed no resistance to pexiganan and PNPs. The very low MIC, low propensity for generating resistance and quick onset of activity are ideal characteristics for potential therapeutics. The *in vivo* pexiganan and PNPs gastric mucoadhesion efficiency and *H. pylori* clearance efficiency were evaluated by administration to rats and repeating oral administration to *H. pylori* infected model mice. The effect of eradication for *H. pylori* was examined by urease tests, and a microbial culture method. The results demonstrated that PNPs adhered to gastric mucosa more strongly and could stay in stomach for prolonged periods for more effective *H. pylori* clearance. In conclusion, pexiganan is a promising anti-*H. pylori* infection agent and when administered in the form of PNPs it more effectively cleared *H. pylori* than pexiganan administered in the form of an suspension, suggesting the utility of mucoadhesive pexiganan nanoparticles (PNPs) to eradicate *H. pylori* infections.
